# Effect of calcanean bone‐tunnel orientation for teno‐osseous repair in a canine common calcanean tendon avulsion model

**DOI:** 10.1111/vsu.13796

**Published:** 2022-03-12

**Authors:** Weston L. Beamon, Daniel J. Duffy, Yi‐Jen Chang, George E. Moore

**Affiliations:** ^1^ Department of Clinical Sciences, College of Veterinary Medicine North Carolina State University Raleigh North Carolina USA; ^2^ Veterinary Administration, College of Veterinary Medicine Purdue University West Lafayette Indiana USA

## Abstract

**Objective:**

To determine the influence of bone‐tunnel anchoring technique on teno‐osseous repair of the common calcanean tendon (CCT) in dogs.

**Study design:**

Randomized, ex vivo, biomechanical.

**Population:**

Forty‐two skeletally mature canine hindlimbs.

**Methods:**

Canine hindlimbs were dissected to produce a model simulating avulsion of the CCT and accessory tendons from the calcaneus. Hindlimbs were randomized to 1 of 3 anchoring techniques (*n* = 14/group): a single transverse tunnel (TT), vertical tunnels (VT), or modified bone tunnels (MT) for teno‐osseous repair in a 3‐loop‐pulley (3LP) pattern using 0 USP polypropylene. Yield, peak and failure loads, construct stiffness, loads to produce a 3 mm teno‐osseous gap, and failure modes were compared between groups.

**Results:**

The only difference detected consisted of TT constructs yielding at loads 25% higher than MT constructs (*P* = .027).

**Conclusion:**

Although yield loads were lower in MT constructs than other groups, the bone‐tunnel anchoring techniques tested here did not appear to influence the biomechanical properties or gapping characteristics of teno‐osseous repairs in this canine CCT avulsion model.

**Clinical significance:**

All drilling techniques and bone‐tunnel orientations tested in the study reported here offer viable options to reattach the CCT to the calcaneus. Surgeons should evaluate how bone‐tunnel orientation may affect placement of adjunctive fixation methods to stabilize the talocrural joint after primary CCT repair in dogs.

## 1 INTRODUCTION

Tendinous injury affecting the gastrocnemius tendon (GT) in dogs is observed to occur at the enthesis, musculotendinous junction, or midbody of the tendon.^1^ Tendon rupture occurring at the enthesis occurs with variable degrees of avulsion of components of the common calcanean tendon (CCT) and can vary regarding patient signalment, chronicity, and level of degenerative tendinous pathology.^1^, ^2^ Complete rupture involving all components of the CCT is seen in 27%‐43% of dogs with this injury. Partial injury and associated rupture with the superficial digital flexor tendon (SDFT) remaining intact occurs in 22%, with injury affecting the GT alone seen in 20% of cases encountered clinically.^1^, ^2^ In dogs, avulsion of the GT and accessory tendon from the calcaneal tuberosity are commonly repaired surgically using a combination of bone tunnels and core suture patterns to oppose the avulsed tendinous tissue to the enthesis of the GT.^3^, ^4^ Debridement or osteotomy of the proximal aspect of the tuber calcanei is frequently employed to remove sclerotic bone, peritendinous osteophyte/enthesiophyte formation and encourage a healthy bone bed to promote teno‐osseous integration.^1^, ^2^, ^5^ Temporary rigid immobilization of the talocrural joint is then typically performed to limit flexion and decrease the strain experienced at the repair site.^1^, ^6^ Despite excellent outcomes frequently cited,^7^, ^8^, ^9^, ^10^ Worth et al.,^11^ reported a 30% complication rate reported following surgical treatment for CCT injury in working dogs.

Limited hindlimb motion and joint stabilization are necessary to counteract the force sustained by the canine CCT during weight bearing, calculated to be ~400 N at the trot in 30 kg medium to large mixed‐breed dogs.^12^ Lister et al., demonstrated that although talocrural immobilization significantly reduced GT strain compared with normal controls, continued isometric gastrocnemius muscle contraction still caused continued strain at the enthesis.^13^ A CCT avulsion model in rats showed that healing at the teno‐osseous junction progressed slowly during the first 4 weeks. This was then followed by 6–8 weeks of rapid healing, tendinous organization, and bony ingrowth, resulting in a 5× increase in strength compared to immediately postoperatively.^14^ Close apposition of the tendon to bone interface, with prevention of gap formation <3 mm during this protracted period of healing is critical to allow progression of osseous integration and tendinous remodeling.^15^ Surgical techniques without use of immobilization for 5 to 9 weeks may predispose repairs to failure.^2^ In dogs, it is also essential that early, controlled exercise and progressive controlled loading be used postoperatively to allow for tendon remodeling, collagenous realignment, and prevention of fibrous adhesions.^16^, ^17^ This suggests a need for repairs that can withstand biomechanical forces independent of immobilization techniques.

Limited information currently exists regarding the influence of teno‐osseous repair methods in dogs. Multiple surgical techniques have been described to anchor suture to the calcaneus.^2^, ^3^, ^4^, ^5^, ^18^ A single bone tunnel drilled in the calcaneus, oriented in a mediolateral direction (transversely), through which suture was then passed is described to secure the CCT to the calcaneal tuberosity.^18^ A double drilling technique was also described in which both respective bone tunnels were drilled in a centro‐medial and centro‐lateral direction, respectively, distal to GT enthesis.^1^, ^17^ A modified tunnel technique was also described by Wilson et al.,^16^ comparing a 3‐loop pulley (3LP) and double Krackow pattern for repair following GT avulsion. To date, there is a paucity of information regarding the effect on bone‐tunnel orientation and anchoring technique for repair of canine CCT and its effect on tissue biomechanics and gap formation at the enthesis. This information is of importance to aid with intraoperative surgical decision making during the drilling and orientation of calcaneal bone tunnels for CCT repair. Information gained from such studies may lead to changes in tunnel orientation and refinements in repair techniques. The objective of this study was to compare the biomechanical properties and gapping characteristics using 3 different drilling techniques and respective bone‐tunnel orientations to anchor the CCT to the calcaneus in a canine model. Our null hypothesis was that there would be no difference in the biomechanical properties or gapping characteristics between different bone‐tunnel orientations used in this study.

## 2 MATERIALS AND METHODS

### 2.1 Cadaver collection

Twenty‐one skeletally mature canine cadavers >1 year of age were obtained from multiple local animal shelters after consented donation. Dogs weighed between 28 and 32 kg and were medium to large breed whose sex was not recorded. Cadavers were acquired within 2 h of euthanasia (1 ml/5 kg IV, Euthasol, Virbac, Fort Worth, Texas) for reasons unrelated to this study. Following euthanasia, a focused orthopedic examination was performed by an ACVS diplomate (DJD) to ensure there was no evidence of orthopedic disease affecting the hindlimbs. This study was exempt from an IACUC protocol by North Carolina State University, Department of Clinical Sciences due to the secondary use of cadaveric tissues. Hindlimb specimens were excluded if there was any stifle instability, angular limb deformity, or musculotendinous pathology evident on examination.

### 2.2 Specimen preparation

Paired hindlimbs were serially dissected to isolate individual components of the CCT. The insertion of the GT and accessory tendon (formed from the musculotendinous contributions of biceps femoris, gracilis, and semitendinosus muscles) onto the dorsocentral and proximomedial aspect of the calcaneus respectively were preserved. The SDFT and surrounding retinaculum were removed to the periosteum using a #10 scalpel blade. Proximally, the SDFT was transected at its myotendinous junction. Distally, the pes was preserved following disarticulation of the talocrural joint. A complete osteotomy through the femur immediately proximal to the femoral trochlear groove was then performed using a reciprocating saw (DeWalt Power Equipment, Anderson, South Carolina). Dissected hindlimbs were then wrapped in saline‐soaked gauze, placed in resealable bags (Ziplock, 1‐Gal, SC Johnson, Mt Pleasant, Wisconsin), and stored at −20 °C. Limbs were thawed at room temperature (21 °C) for 12 h prior to testing.^19^ Thawed limbs were then randomly assigned to 3 groups using computer software (https://www.randomizer.org) with *n* = 14 hindlimbs/group. Limbs originating from the same cadaver were controlled from being placed in the same experimental group.

### 2.3 Surgical repair

**FIGURE 1:**
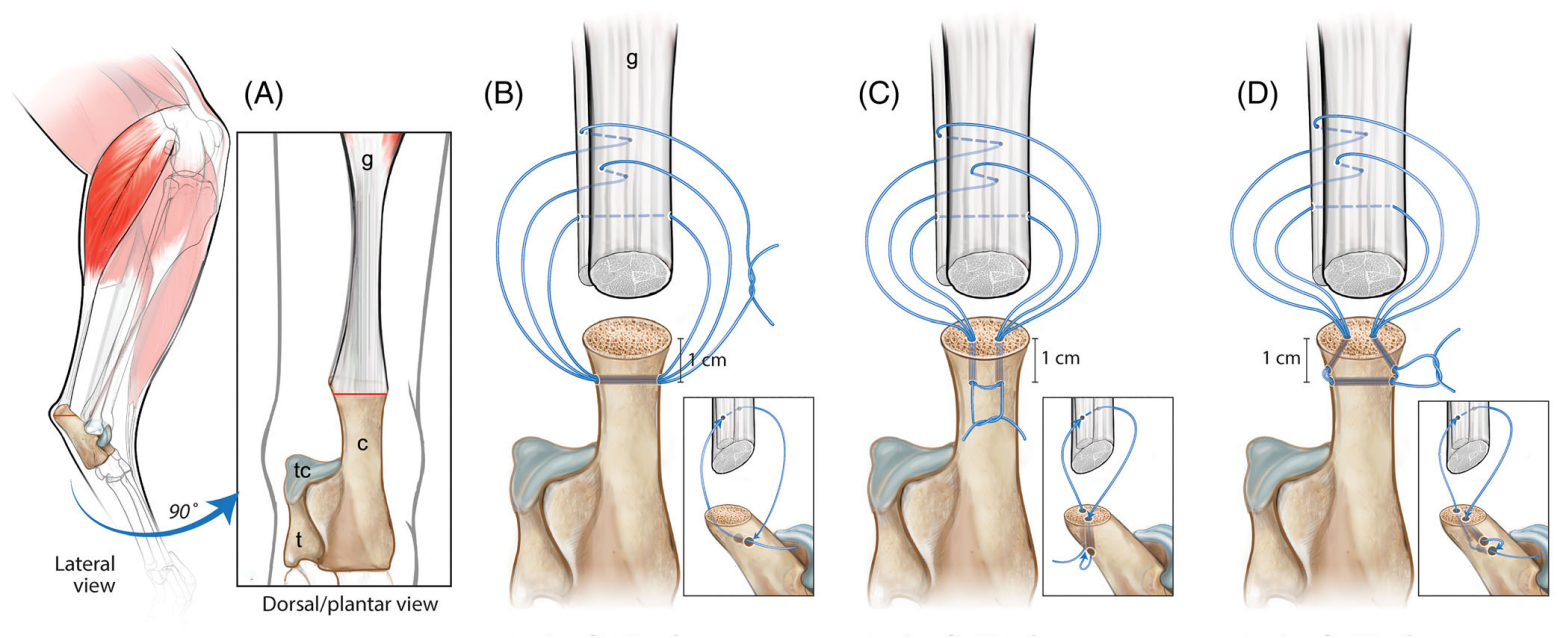
(A) Illustration of the transverse calcanean osteotomy from the point of the caudo‐distal calcanean sulcus to a distance of 5 mm craniodistal to the calcanean tuberosity. Insert: relative orientation of the gastrocnemius tendons (G) and accessory tendon onto the dorsocentral and proximomedial aspect of the calcaneus (C) respectively. Insert is shown representing a dorsoplantar view, medial (M) and lateral (L) are labeled at the bottom of the image. (B) Illustration of a transverse tunnel (TT) construct consisting of a single mediolateral 2.0 mm bone tunnel, 1 cm distal to the center of the calcanean osteotomy, and equidistant from the cranial and caudal calcaneal bone margins. (C) Illustration of a vertical tunnel (VT) construct utilizing 2 parallel, equidistant 2.0 mm bone tunnels beginning at the osteotomy site and exiting 1 cm on the caudodistal aspect of the calcaneus (D) Illustration of a modified bone tunnel (MT) construct consisting of a 2 mm transverse bone tunnel and 2 × 1.5 mm divergent bone tunnels drilled in a centro‐medial and centro‐lateral direction and exiting 2 mm distal to the tibial tuberosity. A 3‐loop pulley (3LP) pattern is used in all repairs with loops placed 60° apart using 0 USP polypropylene. Sequential loops are placed at a distance of 5, 10, and 15 mm from the calcaneal osteotomy site respectively. Abbreviations: g: Gastrocnemius; c: Calcaneus; t: Talus; tc: Talocrural joint

All surgical repairs were performed by a trained senior veterinary student (WLB), closely observed by a board‐certified small animal surgeon (DJD). At the level of the enthesis, the GT and accessory tendon were transected using a #10 scalpel blade creating a complete transverse tenotomy immediately proximal to the calcaneal tuberosity. Following tendinous transection, the pes was held within a mechanical vice and an oscillating bone saw (DePuy Synthes, Palm Beach, Florida) was used to perform a calcanean osteotomy from the point of the caudo‐distal calcanean sulcus to a measured distance of 5 mm craniodistal to the calcanean tuberosity (Figure 1A – red line). The osteotomy was performed in a mediolateral plane, generating a flat surface to mimic a calcaneal osteotomy as would be performed during clinical cases. While positioned within the mechanical vice, bone tunnels were drilled according to their randomly assigned group using heat‐treated chromium stainless steel drill bits (DePuy Synthes, Palm Beach, Florida). A metric ruler (Medline, Northfield, Illinois) was used to ensure consistency for points of drill bit entry and exit respectively. The transverse tunnel (TT) constructs consisted of a single 2.0 mm bone tunnel drilled in a mediolateral direction, 10 mm distal to the center of the calcaneal osteotomy, and equidistant from the cranial and caudal calcaneal bone margins (Figure 1B). The vertical tunnel (VT) constructs utilized 2 parallel 2.0 mm bone tunnels. Bone tunnels were drilled equidistant from one other with the calcaneus orientated perpendicularly, starting at 33% and 66% of the measured (mediolateral) diameter of the calcaneal osteotomy surface that exited 10 mm on the caudodistal aspect of the calcaneus (Figure 1C). The modified bone tunnel (MT) construct repairs were performed as previously described,^6^ initially using a 2.0 mm transverse bone tunnel as described for the TT group. Following drilling of the TT tunnel, 2 × 1.5 mm divergent bone tunnels were drilled in a centro‐medial and centro‐lateral direction at a measured distance of 33% and 66% of the width calcaneal osteotomy site respectively. The medial and lateral exit points of the transverse tunnel were 2 mm proximal to the TT, thus creating a triangular configuration that was then used for subsequent suture passage (Figure 1D). The pes was then removed from the vice, placed on a cutting board, and tendons were repaired ensuring anatomic alignment of the CCT musculotendinous units. In all constructs, tendon reattachment was performed using 0 USP polypropylene suture (Surgipro, Medtronic, Mansfield, Massachusetts). Each suture strand was removed from the packaging and the swaged‐on needle removed, with the suture cut at a ~45°. The lumen of a 20 gauge hypodermic needle (Monoject, Cardinal Health, Dublin, Ohio) was used to facilitate suture passage through the respective bone tunnels. In TT constructs, suture was passed through the transverse bone tunnel in a latero‐medial fashion through components of the CCT then back through the transverse bone tunnel (Figure 1B). In VT constructs, suture was passed disto‐proximal through the lateral calcaneal tunnel, through components of the CCT, then in a proximodistal direction through the medial tunnel (Figure 1C). In MT constructs, suture passed lateromedial through the transverse tunnel, continued through the medial angled tunnel, then through components of the CCT, and finally passed through the lateral angled tunnel (Figure 1B). Suture orientation through respective bone tunnels remained consistent during pattern completion. A modified 3LP pattern was used for all repairs with loops placed 60° apart from the point of initial tendon penetrance with sequential loops placed at a measured distance of 5, 10, and 15 mm from the calcaneal tuberosity.^18^ Sutures were subjectively tensioned to remove any slack from the suture loops while closely opposing the distal cut surface of the tendon to the calcaneal osteotomy ensuring bunching of the tendon did not occur. A square knot followed by 3 additional throws was used and suture cut 3 mm from the knot.

### 2.4 Biomechanical testing

**FIGURE 2:**
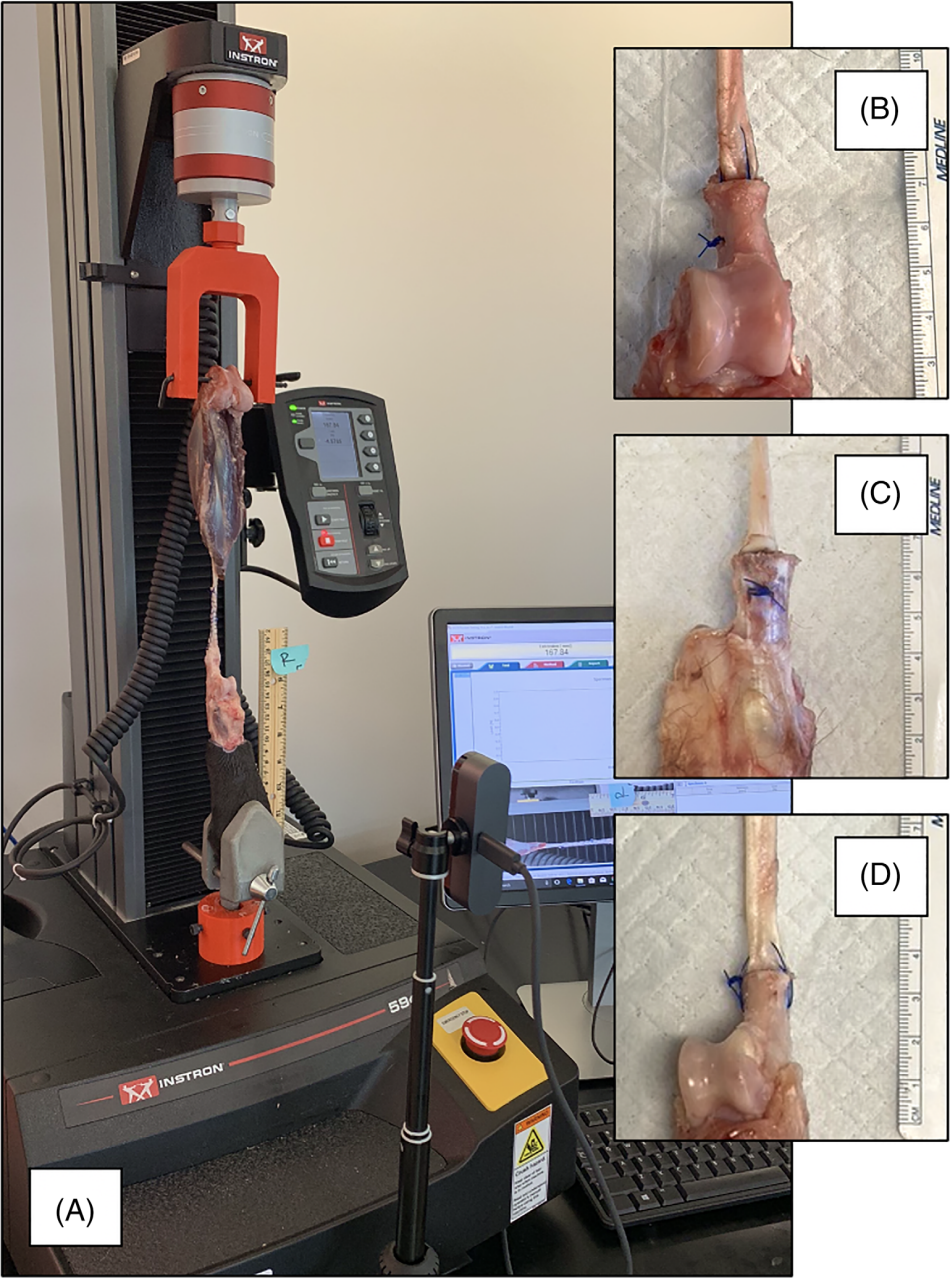
Photograph of the biomechanical testing apparatus with a musculotendinous specimen loaded within the custom testing jig (A). Insert: magnified photograph of tenotomized specimens repaired using 1 of 3 different drilling techniques and bone tunnel orientations. Additional photographs are of different anchoring techniques consisting of modified tunnels (B), vertical tunnels (C), or a single transverse tunnel (D) for teno‐osseous repair to the tuber calcanei using a 3‐loop‐pulley (3LP) pattern in 0 USP polypropylene

Repaired tendons were serially loaded into the biomechanical testing apparatus (Instron, Norwood, Massachusetts). A 4.0 mm tunnel was drilled across the femoral condyle to facilitate proximal attachment of the bone to the machine's crosshead. Distally, the pes was secured using a clamp (SKU‐1652‐1; Sawbones, Vashon Island, Washington) (Figure 2). The calcaneal osteotomy was positioned to orient the cut surface of the bone perpendicular to the base of the testing machine at the start of each test, so the direction of applied load was axially aligned with the teno‐osseous repair. Surgical repairs were then preloaded to 2 N to achieve a consistent resting length and remove any slack from the musculotendinous specimen. Repairs were distracted at a rate of 20 mm/min with data collected at a frequency of 100 Hz until the time of failure identified by a drop of >50% in the applied load. Evaluated biomechanical parameters included assessment of yield, peak, and failure loads (newtons, N), displacement (mm), and stiffness (N/mm). A software program (Matlab R2018b, Mathworks, Natick, Massachusetts) was used to calculate outcome measures of interest by a single investigator (WLB). Yield load was defined as the load at which the first deviation from linearity >5% occurred in the load displacement curve (LDC) noting a change from the elastic to the plastic nature of the construct. The peak load was defined as the greatest load experienced during each test. The failure load was defined as the load applied prior to a sudden acute decrease (>50%) in the LDC or the point of construct failure. Finally, construct stiffness was defined as the extent to which repaired constructs resisted deformation when a load was applied, calculated at 60%‐80% of yield load over the elastic region of the LDC. The mode of failure was recorded during specimen testing (WLB). A high‐definition camera (Panasonic, Newark, New Jersey) recorded each test at 50 frames/s that was synchronized following the defined preload being reached using an automatic trigger system. Gap formation was assessed using the minimum distance (millimeters) at the center of the CCT to assess for development of a 3 mm gap. As shown by Gelberman and Winters, gap formation of >3 mm leads to delayed healing times, reduction in repair site strength with gapping >3 mm increasing the risk for repair failure at the surgical site from 21–42 days following surgical intervention.^15^ Digital calipers were calibrated (ImageJ, NIH, Bethesda, MD) against the ruler of known length placed within the video image. The exact time points at which 3 mm gaps were visualized at the repair site were cross‐referenced with the machine load data, to calculate the loads at which 3 mm gap formation occurred. If failure occurred prior to the formation of an identifiable gap, then this was recorded as “no gap.”

### 2.5 Statistical analysis

A pilot study was performed to refine the study methodology and bone‐tunnel drilling techniques in 6 cadavers (12 limbs) that were not included in the final model. An *a priori* power analysis determined that a sample size of ≥13 tendons/group would provide an 80% power to detect a difference of 30 ± 8 N at a 5% alpha error rate across independent measures. Data were assessed for parametric distribution using the Shapiro‐Wilk test for normality. Continuous variables were normally distributed and described using mean ± SD. Differences in group means were assessed using a mixed linear model with dog/cadaver as a random effect and bone‐tunnel orientation group as a fixed effect. Pairwise comparisons of least square means were conducted using the Bonferroni adjustment for multiple comparisons. A Fisher's exact test was used to compare proportional distributions in 3 mm gap formation and mode of construct failure among experimental groups. All analyses were performed using commercial software (v.9.4, SAS Inc, Cary, North Carolina). A *P*‐value <.05 was considered statistically significant.

## 3 RESULTS

All surgical tenorrhaphies and biomechanical testing were completed without observed technical or procedural error. A total of 42 hindlimbs were tested with no tendons rejected due to defects affecting the bone, muscle, or tendon units. Left and right hindlimbs were equally distributed among all groups (*P* = .87). No specimens were excluded during acquisition or biomechanical testing. Yield load differed by group (*P* = .048). Constructs in the TT group had greater yield loads (25.4%) compared to the MT (*P* = .027), but not VT constructs (*P* = .873). The VT constructs yielded at higher loads (19.6%) compared to the MT constructs (*P* = .038). No difference was observed between groups regardless of bone‐tunnel orientation on peak loads (*P* = .535), failure loads (*P* = .500), or construct stiffness (*P* = .685). (Table 1).

**TABLE 1:** Mean ± SD yield, peak, and failure loads for canine calcaneal bone tunnel orientation groups (*n* = 14/group)

Bone tunnel orientation	Yield load (N)	Peak load (N)	Failure load (N)	Stiffness (N/mm)
Transverse	166.37 ± 36.65^b^	174.99 ± 31.63^a^	174.84 ± 31.67^a^	6.02 ± 1.01^a^
Vertical	158.73 ± 34.85^b^	173.30 ± 28.76^a^	173.30 ± 28.76^a^	5.61 ± 1.15^a^
Modified	132.68 ± 39.76^a^	165.63 ± 22.98^a^	165.00 ± 23.35^a^	5.75 ± 1.22^a^

*Note*: Significant differences between groups in a column are shown using different superscript letters (*P* < .05). Abbreviations: N, newtons; mm, millimeters.

Bone‐tunnel orientation did not affect the loads required to produce a 3 mm gap with no difference seen between groups (*P* = .347). No difference was observed in the frequency of 3 mm gap formation regardless of group (*P* = 1.00) (Table 2). Four constructs failed prior to identification of a 3 mm gap, however, did not differ between groups (*P* = .820).

**TABLE 2:** Number of constructs (n) and proportion (%) of specimens with loads (in newtons) (N) to cause the occurrence of a 3 mm gap between the distal tendon end and calcaneal osteotomy. Superscripts denote significant differences between groups (*P* < .05) n = number of specimens; N = newtons

Tunnel orientation	3 mm Gap formation	
	n (%)	Force (N)
Transverse	12/14 (85.7%)^a^	143.87 ± 19.86^a^
Vertical	13/14 (92.9%)^a^	127.23 ± 27.05^a^
Modified	13/14 (92.9%)^a^	135.08 ± 31.89^a^

Two modes of failure were observed during testing of repaired constructs and included suture breakage (*n* = 14/42; 33%) and suture pulling through the tendon (*n* = 28/42; 67%). No sutures failed at the level of the knot. The occurrence of suture pull through was TT 11/14 (79%), VT 9/14 (64%), and MT 8/14 (57%), and was the most predominant mode of failure seen in this study. No difference was detected between bone tunnel techniques regarding mode of construct failure (*P* = .602).

## 4 DISCUSSION

No effect of bone‐tunnel orientation on the biomechanical properties or gapping characteristics of repaired constructs was observed despite differences of <25% in yield loads seen in the MT group. All methods examined in this study represent viable options for anchoring the CCT through reattachment to the calcaneal tuberosity following CCT avulsion in dogs.

Comparison of different tunnel orientations revealed no effect on the biomechanical characteristics of CCT avulsion repairs apart from a small decrease in yield load seen in the MT group. Although the effect of bone‐tunnel orientation as a primary outcome measure has not been directly assessed, researchers have described the use of transverse, vertical and divergent tunnel orientations for the purpose of GT reattachment following tendinous avulsion.^1^, ^6^, ^20^ Degenerative pathology in the distal CCT, sclerosis of the calcaneal tuberosity, close proximity to the SDFT, and peritendinous osteophyte formation may influence drilling orientation and technique intraoperatively in clinical cases.^1^, ^2^ The use of TT and MT as compared to the VT did not require such extensive dissection and mitigated the need for SDFT elevation, as when intact, this tendon courses over the dorso‐caudal aspect of the tuber calcanei.

The superiority of the 3LP compared to single and double locking loop (LL) patterns is well documented, and the pattern is commonly used clinically for CCT repair in dogs.^12^, ^18^, ^20^, ^21^ Although no difference in the biomechanical characteristics between bone‐tunnel orientation techniques was observed, loads to construct failure were greater than those previously reported by other investigators.^6^, ^20^ These observed results were likely related to differences in suture material and size of suture used.^20^ Use of larger sutures such as size 0 and 2–0 USP have increased tensile strength and improved the biomechanical properties of flexor tendon repairs compared to 3–0 to 5–0 nonabsorbable monofilament suture.^22^ Although subjectively assessed, when load was applied to the tendon, the use of the VT and MT created a more uniform and well apposed anastomosis between the distal CCT and the calcanean osteotomy surface. In TT constructs, upon incremental loading of the construct during removal of suture slack, distortion of the CCT at the tendon‐bone interface without gapping at the teno‐osseous junction was observed, which may affect union and ingrowth clinically.

The GT and accessory tendon have a combined tendinous width that is smaller in size than the corresponding enthesis upon the calcaneal tuberosity in nonpathologic canine CCT. Moores et al.,^12^, ^18^ used 2–0 USP polypropylene and similarly found that the TT constructs lead to divergence of suture material, which may have created this observed suboptimal tendon apposition at the tendon‐bone interface. When tension is applied to a repaired construct using a 3LP pattern, sutures glide through the tendinous tissues and bone in a pulley‐like fashion so that equal load is distributed by each of the 3 loops.^12^, ^18^, ^21^ The 3LP uses 6 suture strands that traverse the tenorrhaphy while LL patterns consist of 2. In contrast, when the LL pattern is loaded, the suture interacts with collagen fibrils as the loops tighten. However, as the LL is loaded, subsequent length is added to the suture strands that bridge the tenorrhaphy, leading to earlier gap formation and separation at the enthesis.^12^, ^18^ This phenomenon may be reduced by tightening the tendon ends; however, this increases bulk at the tenorrhaphy site. Tension redistribution occurs more easily if each loop is tightened sequentially during pattern completion and the ends of the tendon are approximated prior to suture knotting.^21^

Bone‐tunnel orientation may directly influence the direction and interaction of the suture in relation to the longitudinal configuration of the tendon's collagen fibers. These changes may have accounted for the differences seen regarding the point of yield in MT constructs. However, suture elongation and greater ability to remove slack from the bone tunnels in these constructs must also be considered as a possible reason for these observed findings compared to TT and VT constructs respectively. Tunnel orientations in the VT and MT constructs were slightly more proximal and axial in relation to the calcaneal tuberosity and may have resulted in superior ability to maintain the direction of applied load when compared to the native tendon. In addition, the uniplanar nature of the vertically orientated suture that then engaged the tendon with subsequent 3LP suture passes in VT and MT constructs may have allowed better alignment of the suture with the osseous tunnels when compared with the TT. The resulting repair method improved tendon apposition perceptibly, which may translate in vivo to superior teno‐osseous ingrowth; however, these speculative hypotheses should be interpreted with caution until evaluation in vivo.

Previous researchers have altered their study methodology due to perceived differences in teno‐osseous apposition and its effect on gap formation.^6^, ^18^ Gap formation of less than 3 mm is desirable due to the negative effects that could otherwise occur during the normal progression of tendinous healing.^15^ In this study, loads to cause the development of a 3 mm gap did not differ among different bone‐tunnel orientation techniques. However, mean loads to cause 3 mm gap formation (135.17 ± 27.04 N) were greater than reported in prior studies by Wilson et al.^6^ (55.85 ± 9.91 N) and Moores et al.^12^ (49.4 ± 2.4), respectively. Although differences in study design make direct comparisons challenging, these findings may have been due to the inclusion of the accessory tendon during CCT repair. In clinical cases of complete CCT avulsion, the most common presentation in predisposed breeds resulting from degenerative tendinopathy, both the GT and accessory tendon are typically affected. During surgery these tendon ends are often visually indistinct from one another, often enveloped in scar tissue and adhered to the surrounding tendon sheath, hence the reason for inclusion in our study. A study directly comparing repair strength of constructs with and without the addition of the accessory tendon into the final repair would allow the contribution of each tendon and its effect on construct strength to be more clearly elucidated.

Loads to create 1 mm gap formation have been evaluated in previous studies.^12^, ^18^ Moores et al.^18^ described the use of 1 mm measurements while evaluating the loads required to initiate gap formation. Pilot studies found that initial gap formation was often asymmetric in TT constructs. This was most likely caused by differential tensioning of individual suture loops through the single TT as 3LP repairs were loaded. This phenomenon was not observed in VT and MT constructs, which was likely due to the relative orientation of the suture strands to collagen fibrils as they emerged from the tuber calcanei. These findings were not evident when evaluating 3 mm gapping as equal tension was borne by individual suture loops of the 3LP. Gelberman and Winters,^15^ measured tendon strength, stiffness, range of motion, and adhesion formation in gaps <1, <3, and >3 mm respectively, and revealed that only gapping of >3 mm resulted in reduction in normal return of strength and stiffness over time. These findings resulted in deferment of 1 mm gap formation as part of assessed outcome measures.

Suture breakage was the cause of construct failure in a third of tested specimens. Suture breakage typically occurs due to abrasions or weakening of suture when interacting with bone edges, surgical instruments, or suture needles.^23^ Careful observation during osteotomy creation, drilling of bone tunnels, and needle‐facilitated suture passage was performed to reduce the incidence of suture damage; however, rubbing and contact between the suture and then bone could represent a possible cause for suture breakage. Overloading of suture resulting in rupture may represent a more likely alternate cause of suture failure. Suture pull through occurred as the predominant mode of failure when 3LP was used for the primary repair pattern, which agrees with findings in prior studies.^6^, ^12^, ^18^ This occurred in two‐thirds of constructs with no difference seen between groups. Suture pull through has been shown to be affected by suture tensioning on repair site, knot tying, suture pattern, suture configuration, suture selection, and the integrity of the anastomosed tissues.^23^ This study used 0 USP suture compared to 2–0 and 2 USP used in previous studies.^12^, ^13^ It is probable that a larger proportion of constructs failed due to suture pull through due to fewer areas of tendon‐suture interaction when using a 3LP pattern where a large amount of suture remains outside the tendon substance after repair completion. Additional loops, different patterns, or adjunct suture patterns may further reduce the incidence of suture pull through.^20^, ^24^, ^25^, ^26^ Studies involving the modification and refinement of suture patterns to anchor the CCT to the tuber calcanei are warranted.

Limitations of this study included its ex vivo design, which cannot accurately reflect the effects of degenerative tendinous pathology or proliferative changes to the calcaneus that may affect the inherent strength of the repair. In dogs with CCT disease, inflammation can lead to poor collagen fibril quality and decreased suture‐holding capacity. In cases of chronic tendinopathy, a large amount of organized scar tissue may form between the distal tendon ends and the calcaneus. This can make identification of “normal/ healthy” tendinous tissue challenging. The VT design requires evaluation and retraction of the SDFT and surrounding retinaculum during calcaneal bone tunnel creation. This study cannot account for this step in the surgical procedure and its effect on surgical time or postoperative hindlimb function in dogs. In clinical cases, degenerative changes to the bone may dictate the desired placement of a calcaneotibial screw or transarticular external skeletal fixation pins. This may indirectly affect drill bit orientation and the resultant location of calcaneal bone tunnels.

Cadavers used in this study were from dogs of uniform breed, size, and weight. In clinical situations, variations in patient size, need for debridement of the calcanean tuberosity, location of tendinopathy, and length of the tendon available for suture purchase may influence the applicability of these results and their implications for teno‐osseous ingrowth. Where concern exists intraoperatively, the use of adjunctive techniques such as autologous tissues, musculotendinous grafts, epitendinous sutures, synthetic mesh, and tendon plating may also be employed.^20^, ^24^, ^25^, ^26^, ^27^, ^28^ These adjunctive surgical techniques likely affect construct biomechanics and dictate the choice of bone‐tunnel orientation.

In conclusion, despite yield loads differing <25% in the MT group compared to other groups, bone‐tunnel drilling techniques, and bone‐tunnel orientations were observed to have no effect on the biomechanical properties or gapping characteristics following teno‐osseous repair in a canine CCT avulsion model. All bone‐tunnel orientations may represent a viable option for CCT reattachment to the calcaneus. Intraoperatively, surgeons should evaluate how bone‐tunnel orientation may affect the use and placement of adjunctive methods to stabilize the talocrural joint following primary CCT repair in dogs.

## CONFLICT OF INTEREST

The authors declare no conflicts of interest related to this report
